# Landscape limits gene flow and drives population structure in Agassiz’s desert tortoise (*Gopherus agassizii*)

**DOI:** 10.1038/s41598-018-29395-6

**Published:** 2018-07-25

**Authors:** Santiago Sánchez-Ramírez, Yessica Rico, Kristin H. Berry, Taylor Edwards, Alice E. Karl, Brian T. Henen, Robert W. Murphy

**Affiliations:** 1 Department of Ecology and Evolutionary Biology, University of Toronto, 25 Willcocks Street, M5S 3B2 Toronto, ON Canada; 2 Department of Natural History, Royal Ontario Museum, 100 Queen’s Park, M5S 2C6 Toronto, ON Canada; 3 CONACYT, Red de Diversidad Biológica del Occidente Mexicano, Instituto de Ecología, A. C., Av. Lázaro Cárdenas, 61600 Pátzcuaro, Michoácan Mexico; 4 U.S. Geological Survey, Western Ecological Research Center, 21803 Cactus Avenue, Suite F, Riverside, CA 92518 USA; 5 University of Arizona Genetics Core, Thomas W. Keating, Bioresearch Building, 1657 E. Helen Street, Room 111, Tucson, AZ 85721 USA; 6 Alice E. Karl & Associates, 19476 County Road 89, Winters, CA 9569 USA; 7 Environmental Affairs, MAGTFTC MCAGCC, Twentynine Palms, CA 92278 USA

## Abstract

Distance, environmental heterogeneity and local adaptation can strongly influence population structure and connectivity. Understanding how these factors shape the genomic landscape of threatened species is a major goal in conservation genomics and wildlife management. Herein, we use thousands (6,859) of single nucleotide polymorphism markers and spatial data from hundreds of individuals (*n* = 646) to re-evaluate the population structure of Agassiz’s desert tortoise (*Gopherus agassizii*). Analyses resolve from 4 to 8 spatially well-defined clusters across the range. Western, central, and southern populations within the Western Mojave recovery unit are consistent throughout, while analyses sometimes merge other recovery units depending on the level of clustering. Causal modeling consistently associates genetic connectivity with least-cost distance, based on multiple landscape features associated with tortoise habitat, better than geographic distance. Some features include elevation, soil depth, rock volume, precipitation, and vegetation coverage, suggesting that physical, climatic, and biotic landscape features have played a strong evolutionary role restricting gene flow between populations. Further, 12 highly differentiated outlier loci have associated functions that may be involved with neurogenesis, wound healing, lipid metabolism, and possibly vitellogenesis. Together, these findings have important implications for recovery programs, such as translocations, population augmentation, reproduction in captivity and the identification of ecologically important genes, opening new venues for conservation genomics in desert tortoises.

## Introduction

A major goal in conservation genetics involves understanding how landscape features influence population connectivity and structure^1,2^. Heterogeneous environments, geographic distance and life-history traits, such as longevity, mating behavior, and potential for dispersal, can affect rates of gene flow across a species’ range. Agassiz’s desert tortoises (*Gopherus agassizii*) are long-lived, have low-motility, and inhabit one of the most arid environments in North America^3,4^. Populations of *G. agassizii* have been assessed genetically^5–10^ since the species was prioritized for conservation^11,12^. One focus has been the redefinition and delineation of recovery units, originally proposed in the *Desert Tortoise Recovery Plan*^12^, based on clustering methods from population genetic data. While some patterns of population genetic structure have been resolved consistently, marked differences in experimental design, numbers of samples, and sampling strategies fostered inconsistent results, in particular with respect to the resolution of the Western Mojave recovery unit^9,10,12^.

Previous studies have recognized the importance of isolation-by-distance (IBD) as a factor modulating genetic connectivity among desert tortoises^9,10,13,14^. IBD is an evolutionary process by which genetic differences between individuals and/or populations increase with geographic distance^15,16^. The main biological assumption is that many organisms have limited dispersal leading to geographically restricted mating. While geographic distance is an important part the landscape, it is insensitive to the environment, which can be an important source of divergence^17,18^. Isolation-by-environment (IBE) and isolation-by-resistance (IBR) are two ways in which the effects of landscape heterogeneity can be measured with respect to genetic connectivity. Because IBR can conflate both IBE and IBD in empirical data^17^, it can be desirable to explicitly test for IBE, together with IBR and IBD, to untangle multiple competing patterns. A means to accomplish this is to treat IBD as the null hypothesis, against which multiple IBE and IBR models can be tested. While IBE models use overall environmental distances, IBR uses environmental friction or resistance as a proxy for probability of dispersal, where lower resistance leads to higher dispersal^16^. Environmental friction can be quantified by the least-cost distance (LCD), which is the path between two points that accumulates less friction and resistance-distance (RD), which uses circuit-theory to simultaneously weigh many possible routes across a landscape^18^.

Landscape genetics models on *G. agassizii* have reported stronger support for IBD than for IBR^13,14^. However, at a fine spatial scale (<100 km^2^), Latch *et al*.^14^ found weak population structure and weak influence of natural (e.g., slope) and anthropogenic (e.g., roads) factors on genetic connectivity in one population in the central Mojave Desert. Over a broader scale, Hagerty *et al*.^13^ used habitat suitability scores from a model of the distribution of desert tortoises^19^ to quantify landscape friction with LCD and RD. Their results suggested distance due to barriers, such as mountains and deep valleys, are major landscape features limiting gene flow. However, their barrier model was not better than the null IBD expectation^13^. Moreover, these landscape genetic studies have relied on Mantel tests to identify explanatory variables, a method which has been heavily critiqued^20–22^. After comparing a suite of popular methods for assessing spatial correlation, Shirk *et al*.^23^ suggested that linear mixed-effect models using maximum-likelihood population effects^24^ and reciprocal causal modeling^16,25^ were among the most consistent methods; these have not been applied yet for desert tortoises. Furthermore, previous studies on tortoises have relied on at most 20 selectively-neutral microsatellite markers, which inform only about random, stochastic changes in allele frequencies. Genome-wide single nucleotide polymorphisms (SNPs), alternatively, are generally more abundant and can potentially inform about adaptive processes acting upon specific alleles^26,27^.

The genome era promises to resolve many conservation genetics issues associated with breadth of data, marker evolution (e.g., neutral vs. selected sites) and scalability^27^. For instance, thousands of selectively neutral markers can accurately estimate effective population sizes (minimum number of genetically viable individuals) and migration rates (frequency of inter-individual gene exchange), both of which are evolutionary measures critical for assessing conservation status. Further, genomic sites under selection can identify adaptations associated with geographic features, adding potential links to the environment^27,28^. Landscape genomics extends the amalgamation of population genetics and landscape ecology (landscape genetics) on two fronts: (1) access to thousands of putatively independent markers across the genome, which should increase analytical accuracy; and (2) access to genetic data that may be subject to evolutionary forces other than drift, such as natural selection and linkage^29^. The distribution of genetic variation across landscapes can reflect intricate interactions between the environment and evolutionary processes affecting population structure and adaptation to local conditions^30^.

The recently published genome of *G. agassizii* provides a unique resource to study the genomic basis of desert adaptations, and factors related to health, disease, and longevity^31^. Likewise, such data can facilitate population genomic analyses by serving as a reference for mapping variants that can be linked to functional regions. Reduced-representation-sequencing approaches, such as double-digest restriction-site associated sequencing (ddRAD-seq), are rapid, reliable, and cost-effective for generating thousands of SNPs across the genome for hundreds of individuals^32^. These approaches may significantly advance evolutionary and conservation analyses for many species^28,33^.

Herein, we report an analysis of ddRAD-seq data comprising thousands of markers for hundreds of Agassiz’s desert tortoises. Our aim is to comprehensively re-assess population structure throughout the species’ range and provide an understanding of how landscape features influence genetic connectivity. We also seek to associate outlier loci with the functions of neighboring genes and model their distribution in an attempt to better understand local adaptation using reverse ecology. Lastly, we anticipate that our results will better inform wildlife management decisions for recovering declining desert tortoise populations^34^.

## Materials and Methods

### Sampling

**Figure 1:**
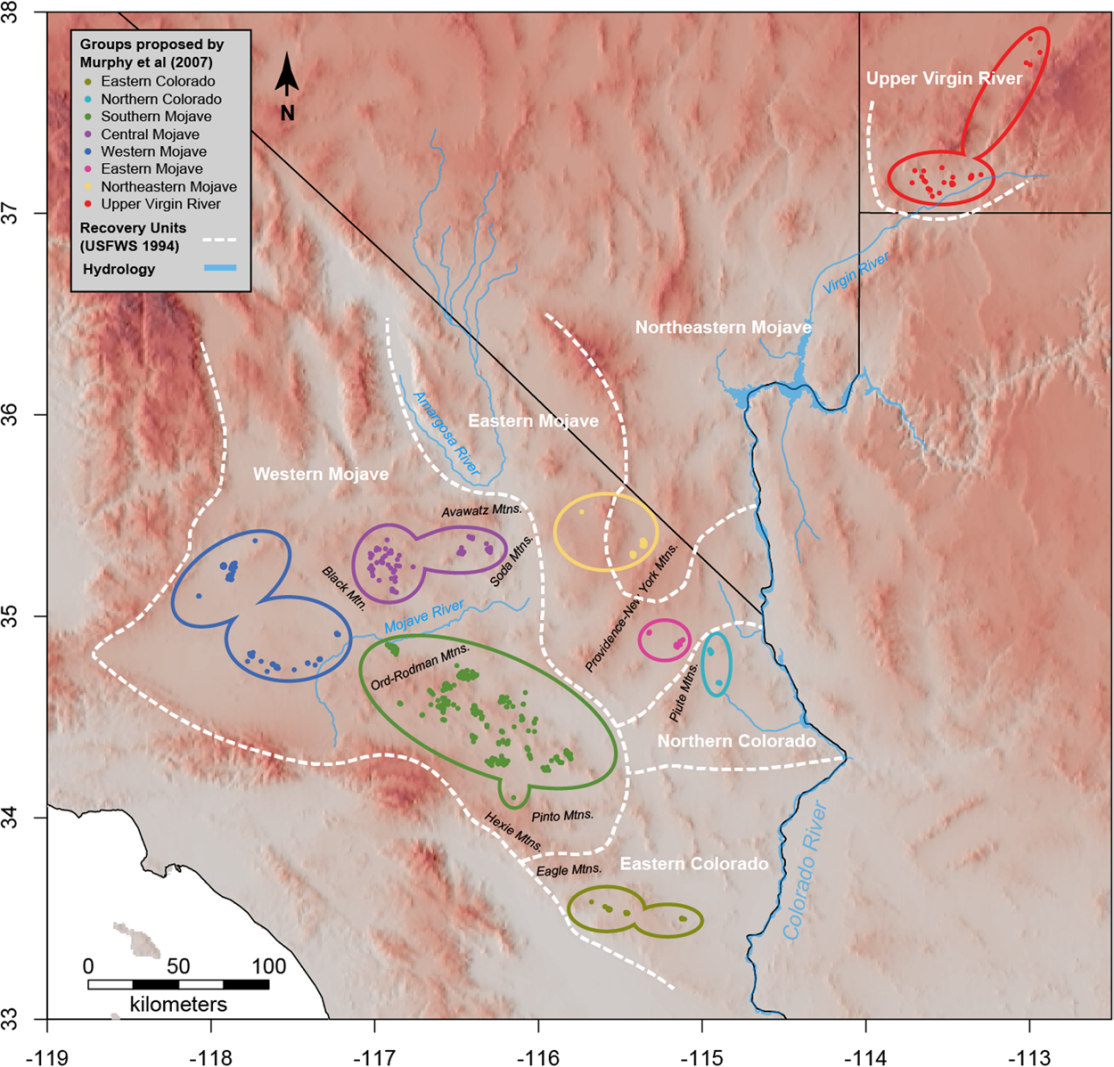
Map of the area with orographic and hydrological details that might act as barriers. USFWS^12^ recovery units are delineated in white. Points represent samples used in this study and are colored by the populations inferred by Murphy *et al*.^9^. The hillshade effect was computed using the *hillShade* function in the R package *raster*^46^ after extracting slope and aspect rasters from elevation data at a resolution of 3.6 arc seconds (USGS, and Japan ASTER Program, 2011, SC:ASTGTM.002:2088835414, 1B, USGS, Sioux Falls, 2011-10-07, downloaded from https://earthexplorer.usgs.gov/). The map was generated with the R package raster v2.6 (https://cran.r-project.org/web/packages/raster/index.html).

We evaluated 538 samples of *G. agassizii* from the Marine Air Ground Task Force Training Command, Marine Corps Air Ground Combat Center, Twentynine Palms, California. These samples came mainly from 23 locations in the southern Mojave region. In addition, we used archival DNA samples employed in Murphy *et al*.^9^ (*n* = 494), which came from hand-captured tortoises whose blood was salvaged from other research projects^7,35–37^. For these, samples were collected at 31 locations across the Mojave and Colorado deserts (electronic Supplementary File S1), except for Nevada and the Beaver Dam Slope in Utah^9^ (Fig. 1). In total, 1032 samples were processed. In the field, animal-handling procedures followed federal and state protocols which adhered to U.S. Fish and Wildlife Service guidelines. Samples were collected under research permits from the California Department of Fish and Game and U.S. Fish and Wildlife Service (TE-06556, TE-17730, BO 8-8-11-F-65R), and complied with the Animal Care and Use Committee of the U.S. Geological Survey and the Animal Research Committee at the University of California, Los Angeles.

### Laboratory procedures and next-generation sequencing

Total genomic DNA was isolated from 50–100 µl of whole blood in 750 µl lysis buffer (50 mM Tris pH 8.0; 50 mM EDTA; 25 mM Sucrose; 100 mM NaCl; 1% SDS) by overnight lysis with proteinase K at 55 °C, followed by robotic extraction using a QIAGEN BioSprint 96 robotic magnetic-particle purification system (Qiagen, Valencia, California, USA) and Invitrogen Dynal bead extraction chemistry (Life Technologies, Carlsbad, California, USA). The recovered DNA was quantified using a BioTEK Synergy HT (BioTEK, Vermont, USA) and diluted working stocks to 5 ng/μl in Low TE (10 mMTris-pH 8.0, 0.1 mM EDTA). Genotyping was performed at the University of Arizona Genetics Core following the protocol of Peterson *et al*.^32^. In brief, genotyping consisted of cutting genomic DNA with two restriction endonucleases (*Sph*I and *Mlu*CI), followed by size-selection, PCR amplification, quantification, and adaptor ligation. Barcode adapters, which recognized individual samples, were ligated to each fragment. Samples were pooled as equimolar concentrations, having 43–48 samples per pool (7 pools per sequencing lane). Libraries were later massively pair-ended sequenced using 4-5 flow-cell lanes with Illumina HiSeq2500 at a read length of 100 bp.

### Bioinformatics

Sequences were retrieved from the University of Arizona Genetics Core server and transferred to SciNet, a high-performance computing server at the University of Toronto. All Illumina pair-end sequence data were filtered for quality control, de-multiplexed (separated and clustered into groups of reads based on individual-level sequence tags) and assembled using *Stacks* v1.44^38^, a software package for restriction-site associated sequencing analysis. Raw reads were initially processed with the program *process_radtags*, filtering all reads with at least one uncalled base (-c option), reads with at least 10% of their length (about 10 bp) having contiguous low-quality bases (<20 Phred score; options: -q -w 0.1 -s 20), and recovering ambiguous barcode tags (-r option). De-multiplexing involved looking for a four-base in-read barcode tag, followed by the restriction site of *sph*I on forward reads (–renz_1 sphI), and the restriction site of *mlu*CI (–renz_1 mluCI) on the reverse end, and the Illumina library index in the FASTQ header (–inline_index). Next, de-multiplexed sequences were mapped (locally aligned) to the reference genome of *G. agassizii*^31^ using *Bowtie2*^39^ and the following settings: -D 15 -R 2 -N 0 -L 20 -i S,1,0.75. The resulting sequence alignment/map file was converted to binary data, sorted, and indexed with *Samtools* v1.3.1^40^. Locus identification, cataloging, and re-matching were performed with *pstacks*, *cstacks*, and *sstacks*, respectively. Only stacks with a read depth of ≥5 (-m in *pstacks*) were kept. For cataloging, loci were determined by genomic position (-g), allowing a maximum of 3 mismatches per sample locus (-n in *cstacks*). A variant-call format file was generated by the program *populations* (*Stacks*) reporting all variable sites per locus (e.g., scaffold/contig); loci found in at least 50% of the samples were retained. In a second filtering step, SNPs with a minor allele frequency below 0.001 were excluded, retaining the SNP with the highest minor allele frequency per linkage group (scaffold) using a Perl script (https://github.com/santiagosnchez/sing_snp_vcf).

### Population structure

We assessed population structure with *Admixture*^41^, which can efficiently handle thousands of SNPs and uses a block relaxation algorithm that accurately estimates ancestry coefficients per individual. To select the best genetic group size (*K*), 10 bootstrap runs were executed using a 5-fold cross-validation (50 cross-validations per *K*) for *K* values ranging from 1 to 10. Only the *K* cluster with the smallest cross-validation error and significantly (Wilcoxon rank sum test) different bootstrapped cross-validation distributions was used for subsequent analyses; other *K* clusters that were only marginally worse were reported. *Q*-matrices were imported into R v3.4^42^ and bar plots with stacked proportions of ancestry per individual were generated.

In addition to *Admixture*, a discriminant analysis of principal components (DAPC)^43^ was performed using the R package *adegenet*^44^. The discriminant analysis of principal components used ordination to graphically depict total genetic variation, maximizing between-group genetic variation, while minimizing within-group variation. Given that the analyses did not allow inclusion of missing data, missing genotypes were substituted for the mean of the available data per locus, where allelic data represented allelic counts separated into two columns (e.g., for a homozygote AA, heterozygote AT, and homozygote TT, the genotypes would be represented as [2,0], [1,1], and [0,2], respectively). DAPC required the *a priori* selection of both the number of retained principal components (PCs) and the number of discriminant functions. Thus, analyses ran a 50-fold cross-validation with data separated into training (90%) and testing (10%) sets (maximum number of retained PCs = 100) using the function *xvalDapc* in *adegenet*. Afterwards, the optimization with the best trade-off between retaining too few or too many principal components was selected. Ordinated genetic distances were then visualized in a scatter plot and colored by genetic cluster in R^42^.

### Spatial interpolation of ancestry coefficients

The *Q*-matrix generated by *Admixture* was used to predict ancestry coefficients on a spatial grid. An R implementation of Kriging interpolation (the *Krig* function from the package *fields*^45^) was applied using a scaling theta of 1, assuming that the unknown covariance was a realization of a Gaussian random spatial processes. To improve model predictions, 200 random locations from outside the predicted distribution were added. For this, a binary grid mask based on the species distribution model (SDM) was generated, which included habitat suitability values above a minimal threshold of 0.2 (based on multiple cross-validation evaluations, see *Species distribution model* below). To exclude internal areas with low suitability scores, the raster was converted to polygons (*rasterToPolygons*) and we used only the polygon with the largest area (electronic Supplementary data S3). Random coordinates of cells outside this polygon were sampled and assumed to have an ancestry coefficient of zero for all groups. Once Kriging models were generated for each group, the *interpolate* function (bilinear method) from the package *raster*^46^ was used to extrapolate ancestry coefficients to the whole surface. Finally, all cells that had ancestry coefficients below the 80% quantile were ignored. This approach was similar (i.e., same under-the-hood functions) to the package *tess3r*^47^.

### Species distribution model

The distribution of *G. agassizii* was reconstructed based on 11 environmental variables that were proposed previously to represent desert tortoise distribution^19^. These variables encompassed topography (elevation and slope), climate (precipitation and temperature), soil (depth to bedrock, fine earth density, coarse fragment volume) and vegetation (vegetation coverage) (Table 1). A detailed variable explanation and plausible connections to tortoise biology and ecology were supplied as electronic Supplementary file S2. All grid/raster data were downloaded at a resolution equal to or higher than 0.01 degrees or 1,000 × 1,000 meters. Raster files with higher resolution were downscaled to 0.01 degrees in R, using the *aggregate* and *resample* functions from the package *raster*. All layers were adjusted to the same coordinate projection system (EPSG: 4326 or +proj = longlat + ellps = WGS84 + datum = WGS84 + no_defs) and cropped to an extent delimited by longitude: −120, −112, and latitude: 32, 38.

**Table 1:** Environmental variables used for species distribution modeling (see electronic supplementary file S2).

Variable (abbrev.)	Ecology	Units	Value range	Source
Elevation (elev)	Topography	m	(−74.91, 4066.31)	ASTER GDEM
Slope (slope)	Topography	radians	(0, 0.52)	*terrain* function in R package *raster*
Absolute depth to bedrock (d2b)	Soil	cm	(0.00, 65155.67)	SoilGrids^106^
Bulk density of fine earth (bd)	Soil	kg/m^3^; depth: 30 cm	(1037.29, 1749.79)	SoilGrids
Coarse fragment volume (cfv)	Soil	percent (%); depth: 30 cm	(0.00, 62.11)	SoilGrids
Wettest quarter mean temperature (Nov–Jan) (bio8)	Climate	°C	(−7.96, 33.20)	WorldClim v2^107^
Driest quarter mean temperature (April–June) (bio9)	Climate	°C	(−4.17 33.01)	WorldClim v2
Wettest quarter mean precipitation (Nov–Jan) (bio16)	Climate	mm	(19.0, 556.3)	WorldClim v2
Driest quarter mean precipitation (April–June) (bio17)	Climate	mm	(0.00, 116.55)	WorldClim v2
Vegetation coverage during the summer (June 2006) (vegS)	VegetationS	NDVI	(−0.20, 0.86)	eMODIS
Vegetation coverage during the winter (Feb 2005) (vegW)	VegetationW	NDVI	(−0.19, 0.93)	eMODIS

Elevation and vegetation data were downloaded from https://earthexplorer.usgs.gov; soil data were downloaded from https://www.soilgrids.org; and climate data was downloaded from http://www.worldclim.org. ASTER: Advanced Spaceborne Thermal Emission and Reflection Radiometer, GDEM: Global Digital Elevation Map, eMODIS: EROS Moderate Resolution Imaging Spectroradiometer, NDVI: Normalized Difference Vegetation Index.

Presence data consisted of 1,848 downloaded georeferenced records of *G. agassizii* from the Global Biodiversity Information Facility (GBIF) server within the area of study. After adding our own georeferenced data, 2,565 presence coordinates were obtained. To avoid data redundancy and same-cell overrepresentation, our presence data were reduced to 645 coordinates representing cells with presence data only (600 × 800 matrix). To generate pseudo-absence data, 10,000 random coordinate points were simulated and cells (1000 × 1000 m) were selected based on those points. Cells with presence data were excluded from the absence data set.

*MaxEnt*^48^ was employed to model habitat suitability based on the environmental data as predictor variables, and presence and pseudo-absence coordinate data. *MaxEnt*, which is a statistical machine-learning model based on the principle of maximum entropy, has been used often for predicting distributions of species^49^. The model was evaluated using a 10-fold cross-validation approach where all the data were separated into small training and testing sets. All statistical evaluation parameters, such as the area-under-the-receiver-operating-characteristic curve (AUC), were summarized across replicates. Variable importance was determined using a jackknife approach. Threshold values were estimated for each replicate and averaged. Habitat suitability scores were inferred using the *predict* function of the package *raster*. Because *G. agassizii* mostly occurs west and north of the Colorado River, we excluded suitability scores east of the Colorado River in the SDM (see electronic Supplementary file S3).

### Geographic- and landscape-based distance matrices

An individual-based approach was used to test for IBD, IBE and IBR. First, genetic distances were estimated with the function *dist_amova* using multivariate genotypic data in the R package *gstudio*^50^. These distances were equivalent to the sum of the squared Euclidean distance between the *i*th and the *j*th genotype^51,52^. Next, matrices with Euclidean geographic and environmental distances were constructed, as well as landscape resistance distances either based on LCD or RD^18^. To have a single individual per cell, one individual was randomly selected in cases where more than one individual was found per cell. Samples from the Upper Virgin River recovery unit (electronic Supplementary file S3) were excluded due to the sampling gap between California and Utah. After this reduction, data for 277 individuals were analyzed. All raster and spatial objects were transformed to Universal Transverse Mercator (UTM) units either using the *projectRaster* or the *spTransform* functions from the R packages *raster* and *sp*. The spatial resolution of all raster grids was 1000 × 1000 m. Euclidean distances were calculated using the R function *dist*, and given that UTM units were meters, Euclidean distances were scaled to km. Data from all environmental layers (Table 1) were proportionally rescaled to fit values between 1 and 10. For environmental distances, we extracted environmental values for subsampled individuals with genetic data (*n* = 277) and for all rasters. Then, we calculated Euclidean environmental distances between individuals.

Expert opinion has been the most common way to empirically assign resistance values. Analytical approaches that involve applying parametric statistical models based on individual distributions have been shown often to be more flexible, informative, and pragmatic^53^. Therefore, we assigned resistance values analytically. First, we extracted the values of cells with presence data (*n* = 1,848) and calculated the density distribution (*density*) for each variable. Then, we fitted cubic smoothing splines (*smooth.spline*) with the values of the density distribution as the response variable and the sampled environmental values from the distribution as predictor variables. We used this model to extrapolate density values to all data cells for each raster. Resistance was assumed to be 1–*density* to assign lower values to cells with higher density or less friction. The cell values in all rasters were again rescaled [1, 10]. We did this for all environmental variables except for the SDM, where direct habitat suitability (1–*suitability score*) was taken as a proxy for resistance; low suitability scores equaled higher resistance. Similarly, slope was evaluated by using both the degree of the slope as a measure of friction and by using the density approach described above. To improve computational efficiency, all 12 layers were trimmed to a polygon defined by the convex Hull of the largest polygon in the SDM (electronic Supplementary file S3).

LCD was calculated using the function *costDistance*, which is based on Dijkstra’s algorithm, in the R package *gdistance*^54^. First, a conductance *transition* object was generated on all layers, considering the eight immediate neighboring cells. Because *gdistance* required transition objects for conductance, rather than resistance values, the function 1/*mean*(*resistance*) was used to obtain a conductance matrix. An R script (https://github.com/santiagosnchez/runCS) that ran *CircuitScape*^55^ in the background was used to calculate RD, directly loading matrices as *dist* objects in R. Least-cost distance and resistance distance matrices were linearized as vectors and stored as data frames.

### Statistical analyses

Following Shirk *et al*.^23^, two approaches for model evaluation were used. The first approach used linear mixed-effect modeling with MLPE^24^. Models were fitted using the function *MLPE.lmm* in the R package *ResistanceGA*^56^. Population assignments (*K* = 5) for every pair of individuals or every distance value in the matrix were used to build the correlation structure. This information was specified as a sparse matrix, which was built using a matrix with two columns and *n*(*n*−1)/2 rows (*n* is the number of individuals), to the ZZ argument in the *MLPE.lmm* function. The model considered the relationships between genetic distances and the predictor distances as fixed effects, and the population structure relationships as random effects. Univariate models were compared using the Akaike Information Criterion (AIC)^23,57^ and Bayesian Information Criterion (BIC) with maximum likelihood, not restricted maximum likelihood (REML), as REML has caused issues in mixed-effect model comparisons^58^. *MLPE.lmm* used the *lme4*^59^ package internally.

The second approach applied reciprocal causal modeling (RCM) with partial Mantel tests^16,25^. The *mantel* function in the *ecodist* package^60^ was used to perform partial Mantel tests for each pair of variables. In every case, the *R*_*m*_ (Mantel’s *R*) was calculated using 999 permutations having one of the variables as the main variable and suppressing the alternative variable (model A). The reciprocal test (model B) was performed next. If the difference in *R*_*m*_ between model A and model B was positive, then the test favored model A, and if it was negative or zero it favored model B^61^. As in Ruiz-Gonzalez *et al*.^61^, *R*_*mA*_−*R*_*mB*_, results were reported in a colored heat map where red colors indicated negative values and blue colors indicated positive values. *R*_*mA*_−*R*_*mB*_ values were summed for each column to simplify variable ranking. The heat map was plotted using the R package *plotly*.

### Outlier loci detection analysis

*BayeScan* v2.1^62^ was used to detect outlier loci (*n* = 646, loci = 6,859). This used a Bayesian multinomial Dirichlet model to estimate allele frequencies and *F*_*ST*_ coefficients, which were then decomposed into population-specific (beta) and locus-specific (alpha) components. Loci for which the locus-specific component was necessary to explain the observed variation were considered non-neutral (e.g., alpha significantly different than 0). Positive alpha values indicated that the locus was under positive diversifying selection, while negative values indicated negative purifying selection. A reversible-jump Monte Carlo Markov chain was then used to select one of two models, in which the alpha component was added or not. Before running the program, a VCF file was converted to the input format required by *BayeScan* for SNP data (https://github.com/santiagosnchez/vcf2bayescan). Next, *BayeScan* was run with default MCMC settings (-n 5000 -thin 10 -nbp 20 -pilot 5000 -burn 50000), except for the posterior odds prior (pr_odds), which was set to 100. Increasing the posterior odds prior increased the sensitivity and made the analysis more conservative, particularly because more than 1,000 markers were analyzed. Outlier loci were detected by plotting the *F*_*ST*_ coefficients against the log_10_ of the posterior odds, and the *q*-values (false discovery rate analog of *p-*value) against alpha. Thresholds were marked by 2 (log_10_(100)) on the x-axis, and *q*-value of 0.05 on the y-axis, for each case. The *closest* function in *bedtools* v2.26^63^ was used to find the closest annotated gene for each outlier locus.

The distribution of the minor allele (lowest frequency allele) of outlier loci was modeled in a similar way as in the spatial ancestry interpolation. A VCF file with outlier SNPs was imported into R using *read.vcf* from the package *pegas*^64^. Then each locus was converted to a numeric multivariate format in which both alleles were separated into homozygotes (1 or 0) and heterozygotes (0.5). The spatial analysis was done as described earlier using Krig interpolation. This analysis was only performed on loci that were close to annotated genes to facilitate physiological interpretation in a spatial context.

### Data availability

Sequencing data were deposited in NCBI under the BioProject ID PRJNA450441. Scripts used for bioinformatics were made available through GitHub (https://github.com/santiagosnchez). All other data were supplied as online supplementary files.

## Results

### Sequence and SNP data

An average of 930,203 reads (SD = 1,335,739, min = 3,098, max = 15,846,603, n = 1032) were generated per individual after quality control filtering. From these, an average of 906,208 reads were successfully mapped to the reference genome. Read depth averaged 13.6 across all samples (SD = 16.8, max = 1049.7). The average number of scored loci was 54,152 (SD = 47,805.54, min = 5,014, max = 342,811, n = 845) per sample after excluding samples with less than 5,000 scored loci. Due to poor data-yield, 386 samples were excluded (good quality, *n* = 646 after exclusion). We catalogued 1,046,121 loci, most of which were excluded for not being present in at least 50% of all samples. Ultimately, after filtering out low quality data, 6,859 SNPs were retained for analyses.

### Genetic structure

**Figure 2:**
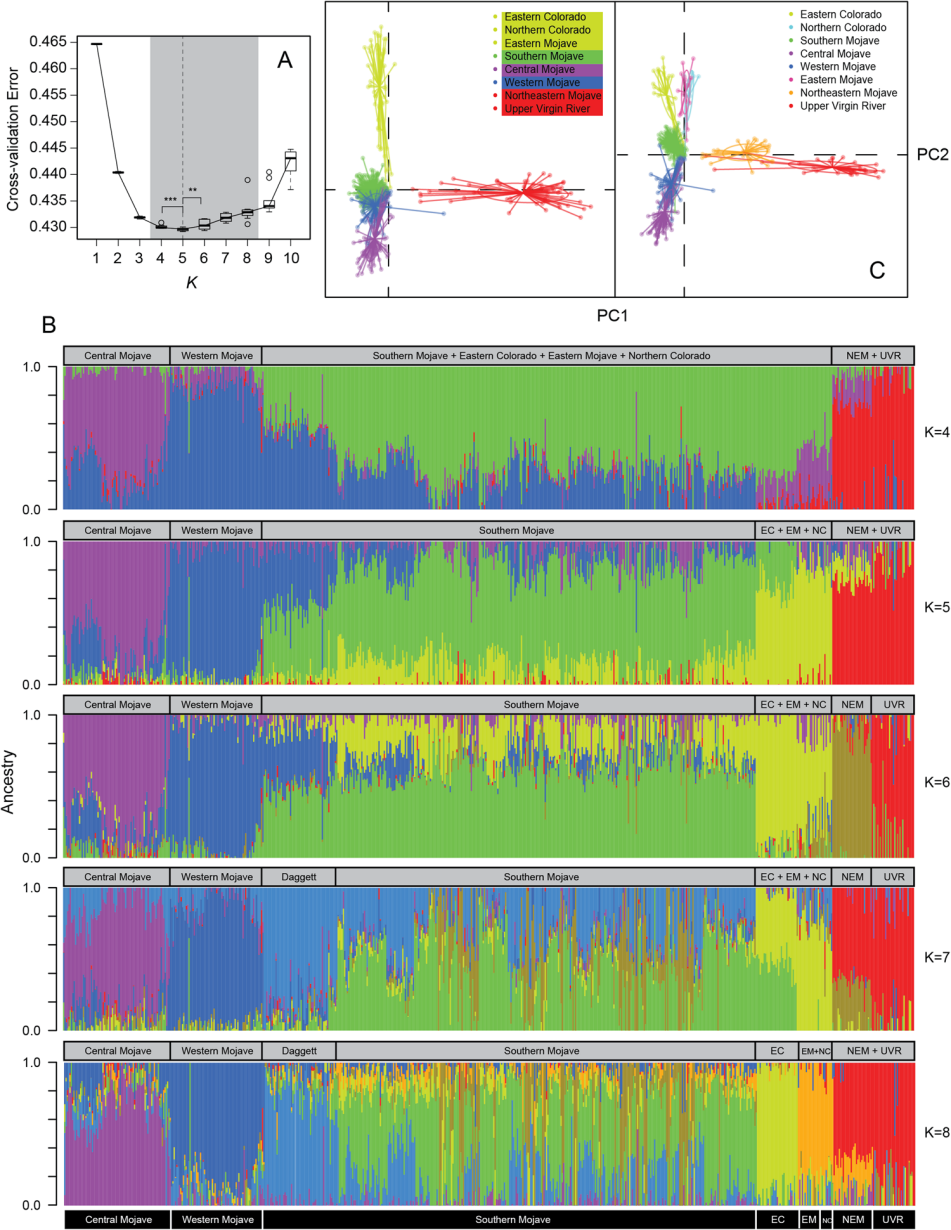
Analysis of genetic structure in Agassiz’s desert tortoise (*Gopherus agassizii*). (**A**) Bootstrapped (n = 10) 5-fold cross-validation error estimations for clusters form *K* = 1 to *K* = 10. The best *K* value is marked with the vertical dashed line. Statistically significant differences were found between the best *K*, and the second and third best *K* values, respectively (Wilcoxon rank sum test: W = 91.5, *p*-value = 0.002 [***]; W = 82,5, *p*-value = 0.01 [**]). (**B**) Bar plot with ancestry proportions per individual for 4 to 8 clusters. (**C**) Genetic ordination analysis using the population structure inferred by Admixture (*K* = 5) and that from Murphy *et al*.^9^ central Mojave (n = 81), western Mojave (n = 71), southern Mojave (n = 374), Eastern Colorado (n = 31), Eastern Mojave (n = 17), Northern Colorado (n = 10), Northeastern Mojave (n = 30), Upper Virgin River (n = 32).

A group size of 5 clusters (*K* = 5) had the lowest cross-validation error (CVE = 0.42897), followed closely by *K* = 4 clusters (CVE = 0.42979; Fig. 2A,B). *K* values ranging from 3 to 9 had marginally worse errors (Fig. 2A,B). For *K* = 5, groups included the following proposed recovery units based on Murphy *et al*.^9^: Cluster 1 (purple) represented the central Mojave group; Cluster 2 (blue) the western Mojave group; Cluster 3 (green) the southern Mojave group; Cluster 4 (yellow) included the Eastern Colorado, Eastern Mojave, and Northern Colorado recovery units; Cluster 5 (red) included Northeastern Mojave and Upper Virgin River recovery units (Fig. 2B). For *K* = 5, pairwise *F*_*ST*_ values ranged between 0.209 (between Cluster 1 and 2) and 0.283 (between Cluster 3 and 5). Recovery units that were recognized with other *K* values included Northeastern Mojave and Upper Virgin River groups (*K* = 6 and *K* = 7) and the Eastern Colorado (*K* = 8) (Fig. 2B). Eastern Mojave and Northern Colorado appeared as a single cluster at *K* = 8 (Fig. 2B). In addition, the population at Daggett, found between the southern, western, and central Mojave groups, was resolved as a distinct group at *K* = 7 and *K* = 8 (Fig. 2B). The DAPC (Fig. 2C) was mostly congruent with the structure found by *Admixture*, as we also found 5 groups. The group representing the most variation in PC1 and the most genetically distant was Cluster 5 (Fig. 2C). Clusters 1–4 differentiated from each other along PC2, with Cluster 4 being the most distinct among them. The most genetically heterogeneous group, with ordination values more centrally distributed, was the southern Mojave (Cluster 3; Fig. 2C). The DAPC based on the *K* = 5 clusters retained the first 30 PCs and three discriminant functions, while the one based on the populations from Murphy *et al*.^9^ retained the first 60 PCs and seven discriminant functions.

### Species distribution modeling

An average AUC of 0.875 (SD ± 0.021) from 10 cross-validation runs indicated a good model fit and a significant deviation from random or homogeneous prediction (i.e., AUC close to 0.5). Based on Jackknife and permutation analyses, the variable depth-to-bedrock (soil) had the largest contribution to the model (average = 42.3%), followed by elevation (topography; average = 15.7%), and mean temperature during the wet season (climate; bio8, average = 13.6%). All other variables contributed from 7 to 1%, with the lowest being coarse fragment volume (soil, Table 1). Visually, the predicted SDM coincided with the Mojave and Colorado deserts. A map of the SDM excluding areas south and east of the Colorado River, and areas with habitat suitability <0.2, was supplied in electronic Supplementary file S3.

### Spatial and landscape genetics

**Figure 3:**
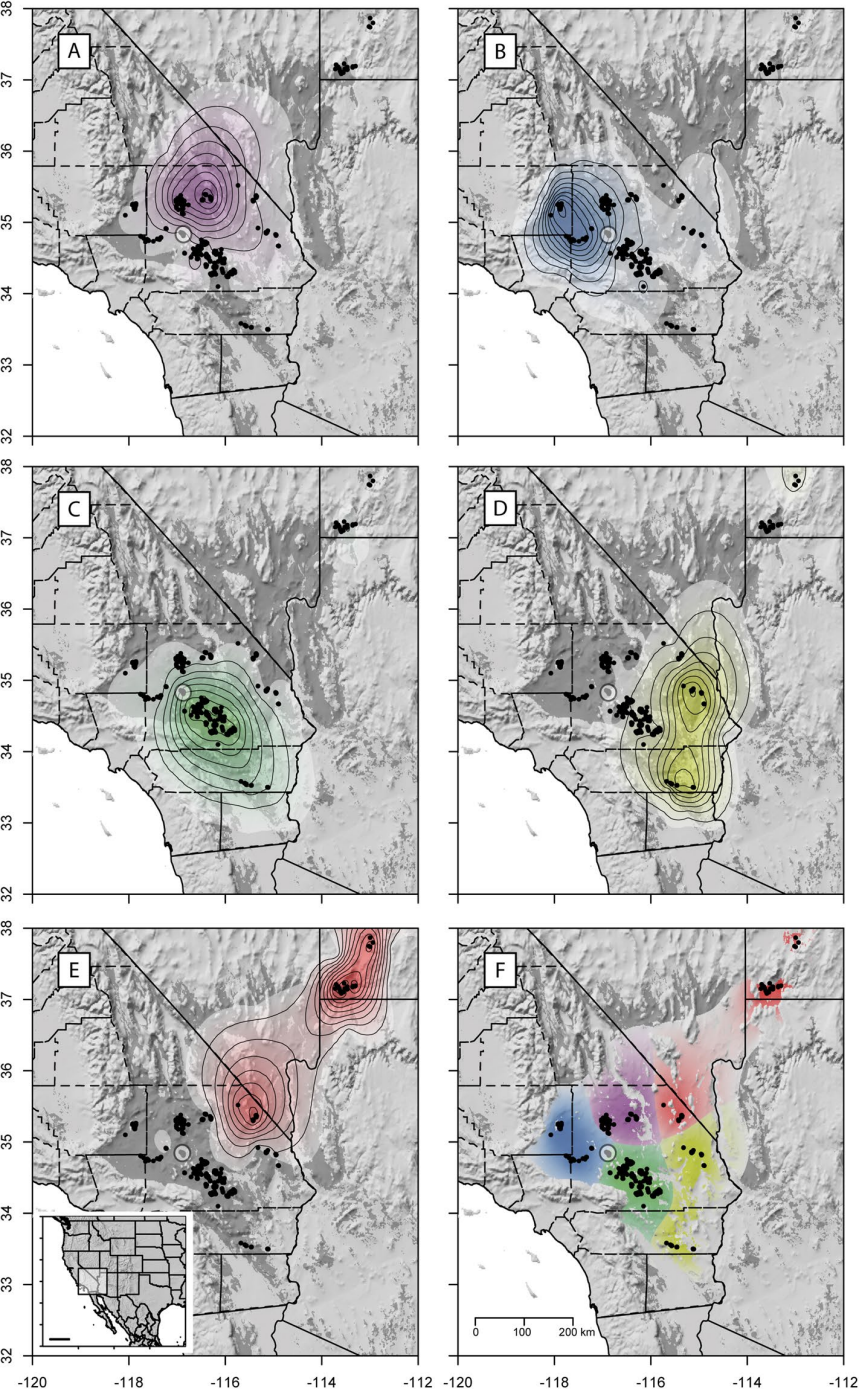
Spatial interpolation of ancestry coefficients of Agassiz’s desert tortoises (*Gopherus agassizii*) using Krig modeling, superimposed on a shaded relief (made with Natural Earth. Free vector and raster map data@http://www.naturalearthdata.com/downloads/10m-raster-data/10m-manual-shaded-relief/). (**A**) Cluster 1; (**B**) Cluster 2; (**C**) Cluster 3; (**D**) Cluster 4; (**E**) Cluster 5. The last map (**F**) combines areas of maximal ancestry proportion for each of the five genetic groups. In F, the total area was trimmed using the species distribution model (darker grey area). Contour lines indicate 0.1–0.9 quantiles. The scale bar in the smaller map in E is equivalent to 500 km. The points highlighted in the white, transparent circle indicate to the population at Daggett. Maps were generated with the R package raster v2.6 (https://cran.r-project.org/web/packages/raster/index.html).

By interpolating the ancestry coefficients, 5 spatially well-defined clusters were identified (Fig. 3F). Most of Cluster 1 was confined to the north-central part of the populations in California (Fig. 3A). Cluster 2 included the far western portion of geographic range (Fig. 3B). Cluster 3 was largely confined to the south-central portion of the Mojave Desert with some presence of admixed individuals (Fig. 3C). Cluster 4 dominated the eastern and southern portions of California, in the eastern and northern Colorado Desert and eastern Mojave Desert (Fig. 3D). Cluster 5 (Northeastern Mojave, Upper Virgin River) stretched from the northeastern portion of the geographic range in California, into Nevada and the southwestern corner of Utah (Fig. 3E).

**Figure 4:**
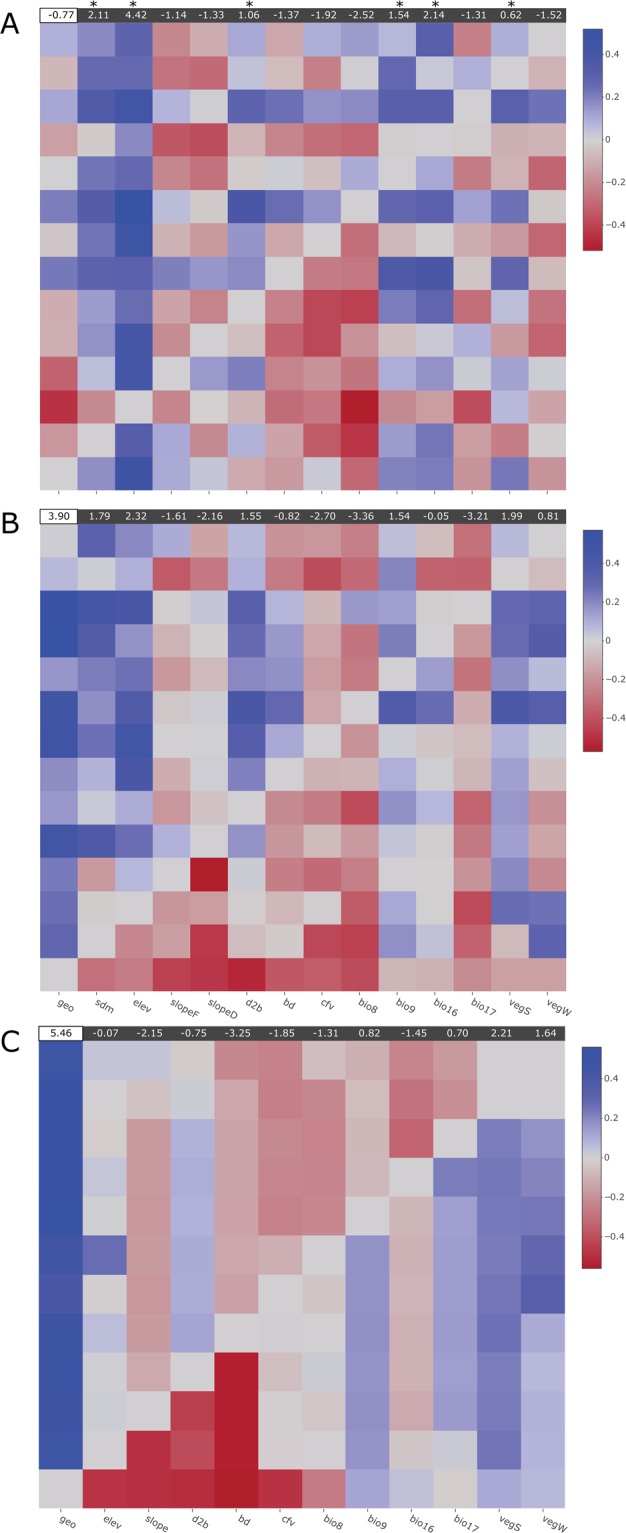
Pairwise heatmaps with reciprocal causal modelling results showing R_mA_ − R_mB_ for (**A**) least-cost distance models, (**B**) resistance-distance models, and (**C**) environmental distance models. Columns represent the main variables and rows represent the alternative variables. Thus, the figure should be read by columns and not by rows. For each column, blue squares indicate a supported variable against an alternative variable. Red squares indicate support for the alternative variable (degree indicated by scale on the right). On top of each heatmap the R_mA_−R_mB_ value is summed for the null model (geographic distance), marked by a white box, and the testing variables marked by a grey box. Variables that were better supported than the null model are also marked with an asterisk.

Both landscape genetic approaches were consistent with each other for the best selected landscape factors. For MLPE, the best model resulted in the mixed-effect linear correlation of genetic distance and LCD based on elevation, followed by average winter precipitation (bio16), habitat suitability (SDM), and depth-to-bedrock (d2b) (Table 2). These variables were better predictors of genetic distance than Euclidean geographic distances (geo) or IBD (Table 2). Similarly, LCD based on variables such as slope (based on slopeD), vegetation coverage (vegS, vegW), the volume of coarse soil fragments (cfv), and the average summer precipitation (bio9) were also better predictors of genetic distance than the null, geographic distance model. In contrast, only one RD variable (summer vegetation coverage) was better than the null model. RCM with partial Mantel tests showed similar results. Six LCD variables (elev, bio16, SDM, bio9, d2b, vegS) had overall better support than the null model (Fig. 4A). RCM did not show strong support for RD models when compared to geographic distances (Fig. 4B). With respect to IBE, none of the environmental distance matrices were better predictors of genetic distance than the IBD or IBR models (Table 2, Fig. 4C). MLPE and RCM differed mainly in the number and relative importance of the best supported predictor variables.

**Table 2:** Mixed-effects linear regression modelling results with maximum-likelihood population effects based on least-cost and resistance-distances.

Distance (model)	Variable	AIC	BIC	logLik	ΔAIC
**LCD (IBR)**	**elev**	**−58881.0**	**−58846.8**	**29444.5**	**0.0**
**LCD (IBR)**	**bio16**	**−58238.2**	**−58204.0**	**29123.1**	**642.9**
**LCD (IBR)**	**sdm**	**−57764.8**	**−57730.6**	**28886.4**	**1116.3**
**LCD (IBR)**	**d2b**	**−57153.7**	**−57119.5**	**28580.9**	**1727.3**
**LCD (IBR)**	**slopeD**	**−57051.7**	**−57017.5**	**28529.9**	**1829.3**
**LCD (IBR)**	**vegS**	**−56945.1**	**−56910.9**	**28476.6**	**1935.9**
**LCD (IBR)**	**vegW**	**−56909.8**	**−56875.6**	**28458.9**	**1971.3**
**LCD (IBR)**	**cfv**	**−56884.0**	**−56849.8**	**28446.0**	**1997.1**
**LCD (IBR)**	**bio9**	**−56881.9**	**−56847.7**	**28445.0**	**1999.1**
**RD (IBR)**	**vegS**	**−56874.3**	**−56840.1**	**28441.2**	**2006.7**
GD (IBD)	geo	−56820.4	−56786.2	28414.2	2060.7
LCD (IBR)	bio8	−56814.2	−56780.0	28411.1	2066.8
LCD (IBR)	slopeF	−56764.2	−56730.0	28386.1	2116.9
LCD (IBR)	bio17	−56702.8	−56668.6	28355.4	2178.2
LCD (IBR)	bd	−56611.6	−56577.4	28309.8	2269.5
RD (IBR)	bio16	−56051.5	−56017.2	28029.7	2829.6
RD (IBR)	sdm	−55851.8	−55817.6	27929.9	3029.2
RD (IBR)	slopeF	−55741.4	−55707.2	27874.7	3139.6
RD (IBR)	bio9	−55692.4	−55658.2	27850.2	3188.6
RD (IBR)	vegW	−55395.9	−55361.7	27702.0	3485.1
RD (IBR)	d2b	−55300.4	−55266.2	27654.2	3580.6
RD (IBR)	bd	−54839.2	−54805.0	27423.6	4041.9
RD (IBR)	elev	−54636.6	−54602.4	27322.3	4244.4
RD (IBR)	bio17	−54552.4	−54518.2	27280.2	328.7
RD (IBR)	cfv	−54189.8	−54155.6	27098.9	4691.2
ED (IBE)	vegW	−54175.1	−54140.9	27091.6	4705.9
ED (IBE)	vegS	−54138.7	−54104.4	27073.3	4742.4
ED (IBE)	bio17	−54111.2	−54077.0	27059.6	4769.8
RD (IBR)	slopeD	−54111.1	−54076.9	27059.6	4769.9
ED (IBE)	bio16	−53332.9	−53298.7	26670.4	5548.1
RD (IBR)	bio8	−53169.2	−53135.0	26588.6	5711.8
ED (IBE)	bio9	−53141.3	−53107.1	26574.6	5739.7
ED (IBE)	elev	−52919.0	−52884.8	26463.5	5962.0
ED (IBE)	cfv	−52901.5	−52867.3	26454.7	5979.6
ED (IBE)	d2b	−52888.9	−52854.7	26448.4	5992.2
ED (IBE)	slope	−52873.2	−52838.9	26440.6	6007.9
ED (IBE)	bd	−52868.7	−52834.5	26438.3	6012.4
ED (IBE)	bio9	−52866.9	−52832.7	26437.4	6014.2

SlopeD stands for ‘slope resistance based on density’ and slopeF stands for ‘slope resistance based on friction’. Rows highlighted in bold indicate models with a better fit than IBD (GD geographic distance) based on AIC and BIC. AIC, Akaike Information Criterion; BIC, Bayesian Information Criterion; logLik, log-likelihood; LCD, least-cost distance; RD, resistance distance; ED, environmental distance; GD, geographic distance; IBD, isolation-by-distance; IBR, isolation-by-resistance; IBE, isolation-by-environment. See Table 1 for details on variable abbreviations.

### Loci under selection

**Figure 5:**
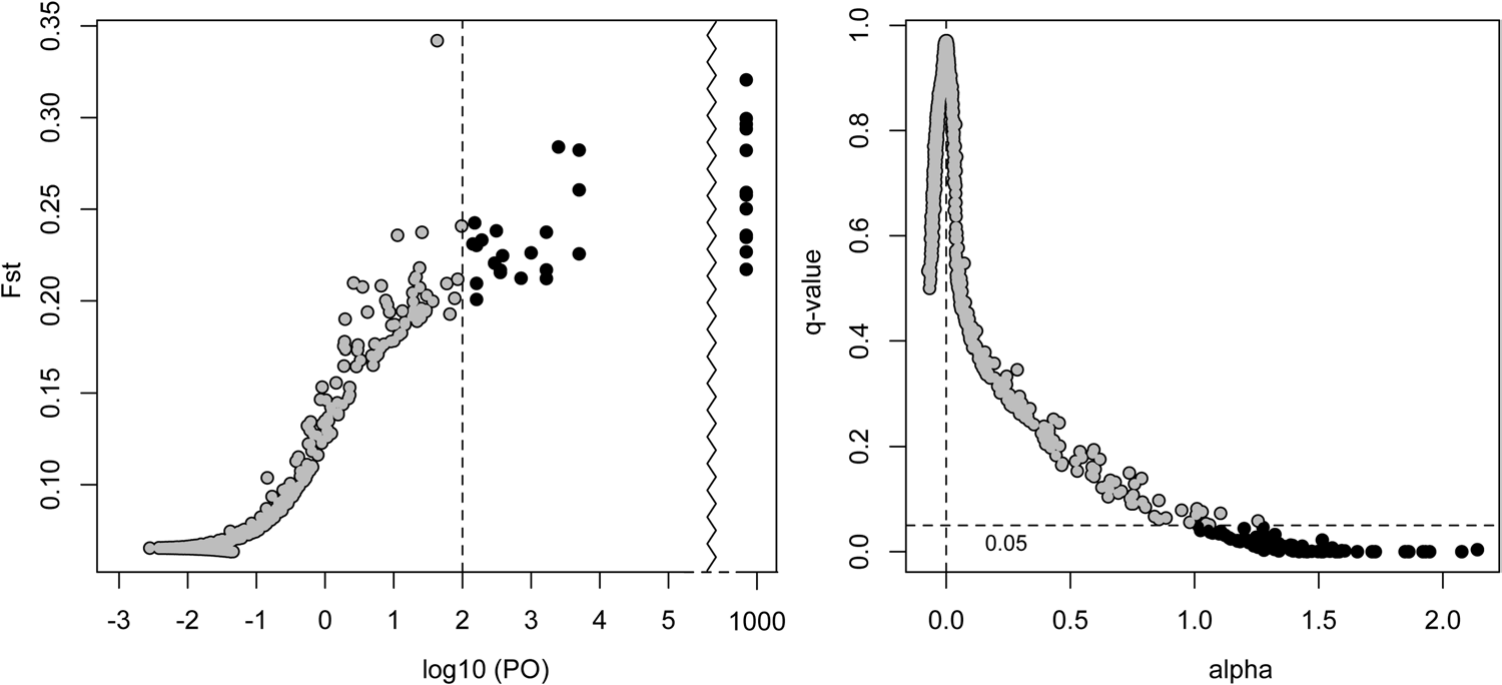
BayeScan outlier loci detection using a log_10_ posterior odds threshold of 100 (left) and a false discovery rate (FDR) threshold of 0.05 (right). Values at 1000 log_10_ posterior odds in the left panel should be infinity because the probability was estimated to be 1; however, BayeScan automatically fixes the log_10_(PO) to 1000 in these cases. All black-colored loci had a probability ≥ 0.99.

Convergence of the MCMC chain in *BayeScan* was assured by plotting the log-likelihood against generations. The prior odds value of 100 was used to select loci with log_10_(PO) > 2, which resulted in loci emitting a very strong to decisive signature (*q*-value > 0.99). This identified 32 outlier loci (Fig. 5A). Eighty-one outlier loci (Fig. 5B) were detected by choosing a *q*-value threshold of 0.05. Given that the first threshold was more conservative, and that the first 32 loci were included in the later 81, we only report the former *BayeScan* results and their closest genes with annotation (if any) in Table 3. From the 32 loci, 21 were on scaffolds with no annotations, 10 were close to an annotated gene of known function, two were close to an annotated gene of unknown function, and the SNP position of five loci occurred within the gene (Table 3). The five SNPs that were found within genes were in introns. The farthest distance from the nearest annotated gene was 67 kbp.

**Table 3:** BayeScan results for 32 outlier loci. Loci are sorted by alpha value showing those with annotation on the top. Distance = 0 indicates variants that occur within a gene

Scaffold	SNP position	loci ID	Gene Id	Annotation	Distance in bp	alpha
scaffold6464	54911	11119_0	00009179	Roundabout homolog 2	60263	1.92
scaffold12629	133684	2293_0	00012395	Cholesteryl ester transfer protein	0	1.73
scaffold22827	31344	5683_0	00018421	Histone H4	5606	1.65
scaffold13833	49357	2747_0	00017165	Thymosin beta-4	36710	1.58
scaffold2756	134627	15060_0	00010622	Protein of unknown function	60624	1.57
scaffold551	55350	15846_0	00017701	SCO-spondin	0	1.53
scaffold15252	24572	3264_0	00018750	Disintegrin and metalloproteinase domain-containing protein 9 (ADAM9)	0	1.53
scaffold2856	267921	7003_0	00010106	Cytoplasmic aconitate hydratase	0	1.52
scaffold19223	291661	4574_0	00009239	Zinc-binding alcohol dehydrogenase domain-containing protein 2	67214	1.50
scaffold2859	55437	15163_0	00012080	Centrosome-associated protein 350	0	1.45
scaffold63	3109	15920_0	00013645	Copine-4	10969	1.45
scaffold491	6232	15794_0	00014935	Protein of unknown function	23631	1.45
C51021930	80	392_0	Not annotated	Not annotated	N/A	2.07
scaffold19860	6644	4787_0	Not annotated	Not annotated	N/A	1.95
scaffold22858	22863	5691_0	Not annotated	Not annotated	N/A	1.93
C52703107	136	690_0	Not annotated	Not annotated	N/A	1.86
scaffold20680	7416	5039_0	Not annotated	Not annotated	N/A	1.86
scaffold11896	12432	1999_0	Not annotated	Not annotated	N/A	1.85
scaffold63199	2272	11018_0	Not annotated	Not annotated	N/A	1.72
scaffold10201	20884	1311_0	Not annotated	Not annotated	N/A	1.71
scaffold3734	10067	8445_0	Not annotated	Not annotated	N/A	1.60
scaffold11541	20785	1856_0	Not annotated	Not annotated	N/A	1.58
scaffold2439	63530	14768_0	Not annotated	Not annotated	N/A	1.57
scaffold8091	75743	12255_0	Not annotated	Not annotated	N/A	1.54
scaffold16925	10756	3814_0	Not annotated	Not annotated	N/A	1.51
scaffold24177	1816	6032_0	Not annotated	Not annotated	N/A	1.51
scaffold3201	8894	7644_0	Not annotated	Not annotated	N/A	1.47
scaffold22935	7321	5705_0	Not annotated	Not annotated	N/A	1.44
scaffold12752	138732	2334_0	Not annotated	Not annotated	N/A	1.42
scaffold7716	3799	12019_0	Not annotated	Not annotated	N/A	1.42
scaffold3736	47634	8451_0	Not annotated	Not annotated	N/A	1.40
scaffold2298	8101	5715_0	Not annotated	Not annotated	N/A	1.34

**Figure 6:**
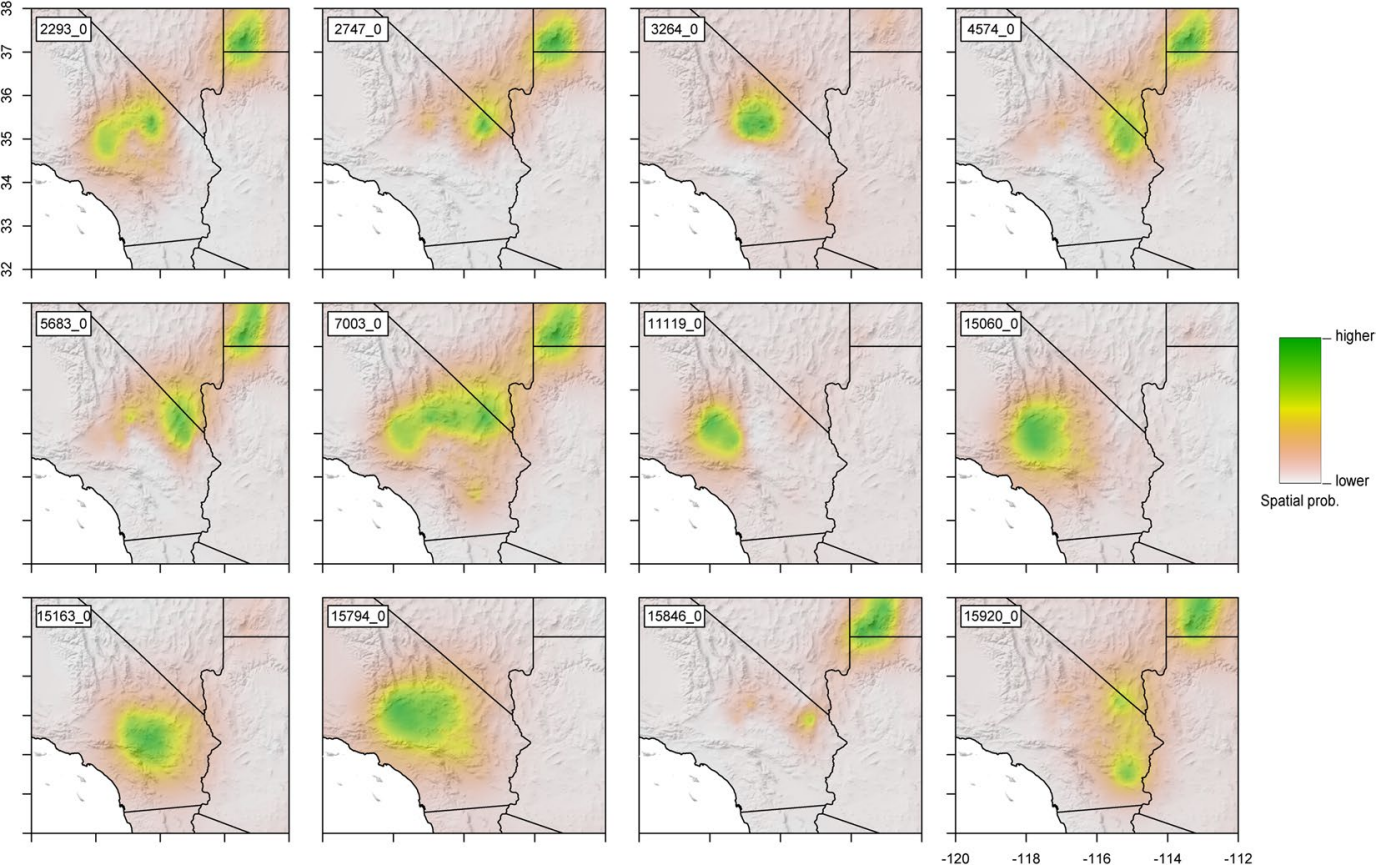
Spatial modelling of outlier minor alleles on or close to an annotated gene. See Table 3 for reference. Raster data is superimposed on a shaded relief (made with Natural Earth. Free vector and raster map data @ http://www.naturalearthdata.com/downloads/10m-raster-data/10m-manual-shaded-relief/). Maps were generated with the R package raster v2.6 (https://cran.r-project.org/web/packages/raster/index.html).

The distributions of the minor alleles of outlier loci that were on or close to annotated genes were modeled (Fig. 6). Overall, the distributions of some loci were fairly population-specific (e.g., 3264_0, 11119_0 and 15060_0 in Fig. 6). Other alleles were found mostly among northern populations (e.g., 2293_0, 2747_0, 4574_0, 5683_0, 15846_0 in Fig. 6), or, to some extent, were rather widespread (e.g. 7003_0 and 15920_0 in Fig. 6).

## Discussion

A major goal in conservation genetics involves performing spatial and genetic assessments of evolutionary significant units to gain knowledge about factors influencing population structure^2,65,66^. The extent to which the landscape limits genetic connectivity may potentially inform about the species potential for dispersal and interactions with the environment^67^. Analyses of vast genotypic data (more than 6,000 SNPs), ample sampling throughout the Mojave and Colorado deserts^68^, and high-resolution GIS data enable powerful insights about the spatial features and genomic composition of desert tortoise populations. The type and volume of data offer unprecedented value for desert tortoise conservation, making possible a more accurate assignment of individuals to spatially defined genetic units, measurement of intra- and inter-population relationships, and allowing for identification of candidate genes on which natural selection may be ocurring^27,28^.

### Species distribution modelling

Species distribution models are an important tool for modern spatial analyses in conservation, and other biological applications^69^. Our SDM constitutes ‘proof-of-concept’ by enhancing multiple analyses. For instance, it improves our spatial ancestry prediction (Fig. 3) where we characterize the absence of genetic data outside a probable distribution margin. We also use the SDM to remove ancestry predicted outside the species’ known range (electronic Supplementary file S3), visually resolving landscape features (e.g. mountains and deep valleys) that may influence spatial genetic structure (Fig. 3F); this step can help us identify likely contact zones between genetic groups. Lastly, we incorporate and test the SDM as a source of heterogeneity in our landscape genetic analyses, as others have done^13^, finding that individual variables can be more informative in explaining genetic connectivity than only using habitat suitability scores.

### Genetic structure

Our analyses detect from four to seven genetically distinct groups (Fig. 2) with well-defined spatial boundaries (Fig. 3) that coincide well with existing and proposed recovery units^9,10,12^. For example, Clusters 1, 2 and 3 (*K* = 5) essentially correspond to the central, western, and southern Mojave populations proposed as recovery units in Murphy *et al*.^9^. Some agreement also occurs with respect to the Eastern Colorado, Northeastern Mojave, and Upper Virgin River populations at *K* = 6 and *K* = 8. To a large extent, our second DAPC analysis (Fig. 1C), based on the populations delimited in Murphy *et al*.^9^, mirrors their 2-dimensional scaling plot using *F*_*ST*_ distances between sampled locations (Fig. 5 in Murphy *et al*.^9^). The high resemblance in results might be partially due to the re-analysis of many of the same samples, albeit using considerably more, new and different data. Analyses of these data show strong genetic relationships within the southeastern (Eastern Mojave, Eastern Colorado, and Northern Colorado), western (western, central, and southern Mojave), and northeastern (Northeastern Mojave and Upper Virgin River) groups.

Our genome-level analyses, together with the results in Murphy *et al*.^9^, support the hypothesis that the Western Mojave recovery unit, as proposed originally in the *Desert Tortoise Recovery Plan*^12^, is a conglomerate of at least three distinct genetic groups. Our results contrast with the study of Hagerty and Tracy^10^, which supports the Western Mojave recovery unit as a single group. In support of our hypothesis, the genetic distinctiveness of these three groups remains consistent at *K* values ranging from 4 to 8 (Fig. 1B), and in the DAPC analysis (Fig. 1C). Other than this disagreement, our analyses are consistent with the Eastern Colorado and Northern Colorado groups described by Hagerty and Tracy^10^. To some extent, our analyses also correspond to their groupings in Nevada and Utah (specifically Southern Las Vegas and Virgin River), which match our Northeastern Mojave and Upper Virgin River groups, respectively. Differences in sampling may account for discordance between our results and Murphy *et al*.‘s compared to Hagerty and Tracy’s. For instance, using stronger sampling in Nevada and Utah, Hagerty and Tracy resolved a higher level of structure than we resolve. In contrast, our study has stronger sampling in the western portion of the Mojave Desert, where we find a higher level of genetic subdivision. Recent genetic simulations on IBD models^69^ demonstrate that population-level sampling (e.g.^9^ and this study) may better resolve membership identification than does random sampling (e.g.^10^) in a IBD scenario. However, we suggest that for conservation purposes all information available should be synthetized into a single framework.

Although we do not quantify gene flow directly, and drift can also be an important factor leading to divergence, both structure and landscape genetics analyses (next section) suggest admixture patterns between the different groups. The southern Mojave population (Cluster 3) seems to be the most admixed group (Fig. 1B,C). Clusters with genetic contributions mainly include geographically contiguous populations, such as those in the western and central Mojave to the west and north, respectively, as well as the Eastern Colorado in the south. Admixture appears to occur between the central Mojave and western Mojave populations, which are close to one another (Figs 2 and 3). While few barriers exist (i.e., Black Mountain in Fig. 1) between these populations, environmental differences are more notorious^9^. Tortoises in the eastern and southern recovery units (Eastern Mojave, Eastern Colorado, and Northern Colorado; as described in^12^; Fig. 1) appear to be more admixed with populations in the southern, central and western Mojave than with populations in the Northeastern Mojave and Upper Virgin River recovery units (Figs 2 and 3). Admixture between the central Mojave group and populations further east may be limited by the Avawatz, Soda, Clark and Mesquite mountains. Similarly, the Northeastern Mojave recovery unit and populations to the south in the East Mojave and Colorado Desert recovery units may be limited by the Providence, New York, Piute and El Dorado mountains in northeastern California and southern Nevada (Fig. 1). Despite the close geographical distance of our samples in the Northeastern Mojave recovery unit to other Californian groups (Fig. 1), this population still has a higher genetic affinity with the more distant Upper Virgin River recovery unit (Fig. 2C,B). This genetic affinity between both groups occurs even in spite of the sampling gap in Nevada, which should result in structured populations^29^.

The extent and directionality of admixture, together with the spatially explicit genetic structure (Figs 2 and 3), suggest a pattern recognizable as IBD. Five previous studies have described IBD as a likely evolutionary force driving population structure in desert tortoises^8–10,13,14^. As the authors suggested, limited dispersal, previous and present barriers, and climatic features are thought to be important factors effecting genetic differentiation^3,9,71–74^.

### Landscape effects on genetic connectivity

The effects of IBD are usually assessed by inspecting relationships between Euclidean (e.g., straight line) inter-individual or inter-population geographic distances and genetic distances^15^. In contrast, more sophisticated landscape genetic approaches^1^ apply IBR models to evaluate the effect of landscape heterogeneity on genetic connectivity^16,18^. Because IBD can conflate IBR, our assessment uses multiple IBE models in a comparative framework. Our results do not find a direct influence of raw environmental distances on population structure. In contrast, IBD and IBR models are consistently better supported than IBE, suggesting that spatial features may be more important than raw environmental differences. Moreover, analyses find that several landscape features are better predictors of genetic connectivity than geographic distances (Table 2, Fig. 4A). Some of these features also may seem to be relevant for niche partitioning between *G. agassizii* and *G. morafkai*, which are genetically close and geographically adjacent species^74^.

Elevation is the best supported variable by both MLPE and RCM, and also contributes significantly to the SDM model. Previous microsatellite-based landscape genetic analyses have concluded that topological features restrict gene flow in desert tortoises^13,14^. Hagerty *et al*.^13^ reported that mountains and deep valleys serve to limit gene flow, albeit with marginal support. This reinforces field observations and distribution models suggesting that tortoises generally avoid steep slopes, high elevation areas, and playas^12,19,75^. Typical desert tortoise habitat can range from sandy plains to rocky or rolling foothills, including alluvial fans, washes, and canyons^12^. Tortoises also spend most of the year underground^76^, which means that they are likely to be found in areas with soils suitable for burrowing or caves in well-developed calcic layers^77,78^. This makes the idea of testing soil variables appealing. In fact, our analyses find support for variables such as the absolute depth-to-bedrock and fraction of coarse (>2 mm) soil fragments (Table 2, Fig. 4A), which might be relevant to burrowing. Depth-to-bedrock also has the highest contribution to the SDM, even more so than elevation, indicating that it is a relevant landscape feature for predicting tortoise habitat^78^.

Rainfall has strong effects on food plant production^79–81^ and provides drinking water essential for life^35,76,82^. Better nutrition and access to water can, in turn, improve health, and increase growth, activity, and reproductive output^35,37,76,82–85^. Rainfall also stimulates growth of plants (e.g., shrubs and other perennials) that provide tortoises shade and shelter, plus stability to soils that support tortoise burrows. Interestingly, our analyses find average winter precipitation and vegetation coverage (winter and summer) to be good predictors of genetic connectivity (Table 2, Fig. 4A). Because tortoises can find suitable habitat conditions at a fine scale^86^, both the scale of the study (1000 × 1000 m) and/or the high variability of the landscape due to low primary productivity of deserts, could have hindered the relationship between vegetation coverage and genetic connectivity. However, our results show that even at broader scales the amount of vegetated land, in particular perennial plants, can have a substantial impact on how desert tortoise populations are structured.

Circuit-based IBR models were introduced as more realistic alternatives to LCP analyses, which assume that individuals have preferred dispersal routes^1,16,18^. In contrast, RD is quantified as the average random walks between locations, assuming that gene flow happens through multiple, alternate routes^18,56^. LCD models for the best variables (Table 2, Fig. 4) are better predictors of genetic distance, which implies that genetic corridors among tortoises tend not to follow random routes. Instead, corridors may follow narrower paths that are optimal for them to increase their movement efficiency. Noteworthy, in nature, tortoises have high site fidelity, and tend not to move far away from burrows, rock shelters and dens. Tortoises are aware of geophagy sites^87,88^, drinking basins^82^ and other resources (e.g., conspecifics) in their home ranges. Thus, because genetic variation accumulates over time, it is important to frame genetic connectivity as a measure that represents evolutionary tendencies of genetic exchange, and not as a measure that represents contemporary movement.

### Management perspective

Understanding the genetic units of tortoises is important for managing this threatened species. Genetic units, among other factors, form the basis of recovery units for the Mojave population of desert tortoises^12^. It is possible to improve management techniques, including population augmentation (e.g., headstarting and translocation), by incorporating knowledge of genetic boundaries and distances that tortoises should be moved. Our analyses delineate genetic population boundaries by using robust sampling for most of the species’ geographic range. These genetic boundaries are similar but not identical to those proposed by Murphy *et al*.^9^ and Hagerty and Tracy^10^.

Averill-Murray and Hagerty^89^, using genetic boundaries drawn by Hagerty and Tracy^10^, wrote that populations “within a 200–276 km straight-line radius of each other (249–308 km measured around topographic barriers) tend to be genetically correlated and may be considered single genetic units for management purposes.”. Our findings, drawing on more robust genetic analyses, indicate the prudence of considering the importance of population boundaries in addition to distance. Distances of 200 km extend across several genetically identifiable populations (i.e., western Mojave, central Mojave, Daggett, and the southern Mojave) in the Western Mojave recovery unit, and across genetically identifiable populations in the southern Mojave to the eastern Colorado Desert. Mixing genetic populations could lead to outbreeding depression, failure to integrate, thrive, and survive^9,90,91^, or outbreeding vigor^90^, although there are no ‘common garden’ studies^92,93^ or other empirical investigations that explore these phenomena for *G. agassizii*. Via conservative management, however, we may limit risks by avoiding population augmentations across genetic population boundaries or over long distances^94^. Consistent with this approach, headstart tortoises in the western, central, southern, and northeastern Mojave genetic populations are being placed within their genetic population of origin.

### Loci under selection and their functions

Our analyses consider outlier loci for two reasons. First, we confirm that the majority (99.5%) of loci are neutral, which is an assumption in models of population structure^95^. Second, the approach can identify genes or neighboring genes of loci that strongly conform to a non-neutral model^26^. In a recent conservation genomics study of the Burmese roofed turtle (*Batagur trivittata*), from about 1500 SNPs, not a single locus departed significantly from neutrality^96^. In contrast, our analyses discover at least 32 loci under potential diversifying selection. Among 12 loci that associate with annotations, five variants occur within introns of five genes (Table 3), upon which the effects of selection are likely to be stronger due to linkage^97,98^, if, in fact, these genes are targets of selection. Analyzing RNA expression of these candidate genes can help understand their fitness effects. Likewise, d_N_/d_S_-type analyses of sequence data for the whole gene and multiple species can also provide additional evidence for selection.

Some genes in the vicinity of these loci have functions that may be involved with neurogenesis, wound healing, lipid metabolism and vitellogenesis. More specifically, noteworthy functions include the following: Beta-thymosin (IPR001152), a multi-function protein involved with cellular processes such as wound healing, actin formation (muscle development), embryonic organ development, and disease pathogenesis^99,100^; *ADAM9* (IPR006586), a membrane-anchored protein involved with cell adhesion, fertilization, muscular development and neurogenesis^101^; Roundabout homolog 2 (IPR032985) and SCO-spondin (IPR030119), which are independently related to axonal migration, growth, neural development, and tissue development^102^; and cholesteryl ester transfer protein (IPR017130), with functions related to lipid and cholesterol control^103^.

The direct relationship between allele frequencies, gene functions, and their implication on the biology of tortoises is difficult to assess. However, the cholesteryl ester transfer protein (IPR017130) might point to interesting future research given the association of lipid metabolism with energy storage and vitellogenesis among desert tortoises^37,85^. Lipid metabolism helps females increase body lipid reserves they use subsequently during periods of low resource availability, such as drought and brumation, and stimulates egg production^85^. Cholesterol and triglyceride levels have been found to vary between females and males, and among seasons, where they are high in spring when females are preparing eggs^37^. Egg production among female desert tortoises varies with the amount of rainfall or concomitant primary production^81,104^ and distribution (i.e., East-to-West Mojave^108^). However, our results show that the minor allele associated with the cholesteryl ester transfer protein occurs mostly in northern areas (Fig. 6: 2293_0) and has no east-to-west variation. Moreover, this protein may serve other functions. For instance, a variant of the cholesteryl ester transfer protein in humans has been linked to larger high- and low-density lipoprotein particle sizes, which may decrease hypertension and cardiovascular disease, therein promoting longevity^105^. A recent comparative genomic analysis in chelonians has revealed several genes involved with longevity and fatty acid metabolism to be under a high rate of molecular evolution^31^.

## Conclusions

Landscape genomics aims at identifying complex interactions between the environment and the genome of individuals in a population. These interactions include, but are not limited, to how the landscape limits gene flow between and within populations, and how genome-wide allele frequencies change as a function of space and the environment. With better inferences about these interactions and underling biological processes, better conservation and wildlife management actions can take place to restore major, threatened species, such as the desert tortoise. For the first time, we generate thousands of genome-wide genotypic data for hundreds of individuals in desert tortoises, which have helped to robustly assess the population structure in the species. By coupling genetic and spatial interpolation techniques, analyses delimit genetic clusters spatially, which can help inform potential locations for translocation and headstart releases, and where interpopulation interactions may occur. We also apply novel statistical methods to evaluate the effect of the landscape on genetic connectivity, using geographical distance as a null model. Our results allow us to build on previous studies, showing how several environmental, climatic, and biotic features explain genetic differences between populations. Finally, we identify potentially non-neutral loci that are in the vicinity of genes that may be involved with neurogenesis, wound healing, lipid metabolism and vitellogenesis. While their direct correlation to the environment is still uncertain, this research opens new directions for conservation genomics in desert tortoises.

## Electronic supplementary material

Supplementary information

Supplementary dataset
