# Supplementary material for: Landscape limits gene flow and drives population structure in Agassiz’s desert tortoise (Gopherus agassizii)
Source: Sci Rep. 2018 Jul 25;8:11231. doi: 10.1038/s41598-018-29395-6 (PMC6060138; doi:10.1038/s41598-018-29395-6)
Supplement: Supplementary file 1 — Supplementary information [file 41598_2018_29395_MOESM1_ESM.docx]

**Landscape limits gene flow and drives population structure in Agassiz’s desert tortoise (*Gopherus agassizii*)**

Santiago Sánchez-Ramírez, Yessica Rico, Kristin H. Berry, Taylor Edwards, Alice E. Karl, Brian T. Henen, and Robert W. Murphy

Supplementary files:

**Supplementary file S1**. CSV file with coordinates, location, and population data for each individual in the analyses.

**Supplementary file S2**. Word document with the description of variables used in the landscape genetics analyses and their potential ties to the biology and/or ecology of desert tortoises.

**Supplementary file S3**. Map of the study area. The area in transparent black was used for trimming environmental rasters. The species distribution model (SDM), indicated as a raster with multiple colors, is trimmed to the 0.2 threshold limit used for spatial ancestry interpolation. Red crosses represent genetic samples from the Upper Virgin River Recovery Unit (UVR), which were excluded from landscape genetic model-testing analyses. White crosses are the remaining genetic samples from California. Scale bar is on the left lower corner. A shaded relief (made with Natural Earth. Free vector and raster map data @ *http://www.naturalearthdata.com/downloads/10m-raster-data/10m-manual-shaded-relief/*) is superimposed and the projection is in Universal Transverse Mercator units (meters E, abcissa; meters N, ordinate; datum WGS 84). The map was generated with the R package *raster* v2.6 (https://cran.r-project.org/web/packages/raster/index.html).

**Supplementary file S1**

Available as an additional CSV file.

**Supplementary file S2**

**Elevation** (meters above sea level): habitat/dispersal friction. Elevation affects numbers of freezing days and therefore amounts of time available for nesting, hatching, feeding, depth of required burrows or dens. Low elevations affect the degree and number of days with very high temperatures and types of vegetation (sparse).

**Slope** (radians): dispersal friction. Steep, precipitous slopes reduce likelihood of movements across some mountain ranges.

**Absolute depth to bedrock** (cm): shelter/burrowing. Affects choice of shelters and burrows. Depth to bedrock is likely to be very high in valleys (where tortoises were once more common) and on alluvial fans but close to the surface on steep slopes and some mountain ranges –thus precluding easy access to shelters.

**Bulk density of fine earth** (g . cm^-3^): shelter/burrowing. Moisture in the soil provides stability to burrows and a cooler shelter during high heat periods. Lower bulk density reflects porous soils that can absorb more water.

**Coarse fragment volume** (%): shelter/burrowing. This is the percentage of soil with particles that are larger than 2 mm. Rocky soils are unsuitable for digging and maintaining a long burrow. If surface is rocky and with larger fragments, tortoises will have more trouble navigating and traveling, especially the juveniles.

**Average temperature during the driest quarter** (April - June, °C): energetics/metabolism. Temperatures during the dry season (e.g., late spring, summer, early fall) are often very high and affect the amount of time the tortoises will spend underground and available above ground for courting and combat. Dry season temperatures also require burrows or dens of sufficient depth and length to avoid exposure of tortoises to high temperatures, loss of stored energy and water.

**Average temperature during the wettest quarter** (Nov. - Jan., °C, range of values in dataset): energetics/metabolism. Following winter rains, tortoises will emerge from shelters to begin the spring activity season (e.g., foraging, egg production, and possibly mating or male-male agonism). Late winter and spring are principal times of the year for tortoises to eat succulent green vegetation, construct nests, lay eggs, and travel. Warm but not hot air and surface temperatures and few or no freezing nights, enhance above ground activity during the wet season.

**Average precipitation during the driest quarter** (April - June, mm): energetics/metabolism/food. If monsoonal rains occur during the dry season, tortoises emerge from underground shelters to drink free water. In the eastern and southern parts of the geographic range, a limited flora will germinate and flower with summer rains, and forage for a secondary feeding period may be available.

**Average precipitation during the wettest quarter** (Nov. - Jan., mm): energetics/metabolism/food. Precipitation primarily occurs in late fall and winter and stimulates growth of forage for tortoises to eat in late winter and spring.

**Vegetation coverage during the winter** (Feb 2005, Normalized Difference Vegetation Index, NDVI): energetics/metabolism/food/habitat. Range-wide, tortoises require succulent, green forbs and herbaceous perennial vegetation following late fall; winter rains support subsequent activity health, physiology, reproduction, behavior, and survival in spring. Late winter and spring are the principal feeding times, except during drought years.

**Vegetation coverage during the summer** (June 2006, Normalized Difference Vegetation Index, NDVI): energetics/metabolism/food/habitat. Germination of summer forbs and herbaceous perennials following summer precipitation can be important secondary food sources in the eastern and northeastern Mojave Desert and in the Colorado Desert.

**Supplementary file S3**

**
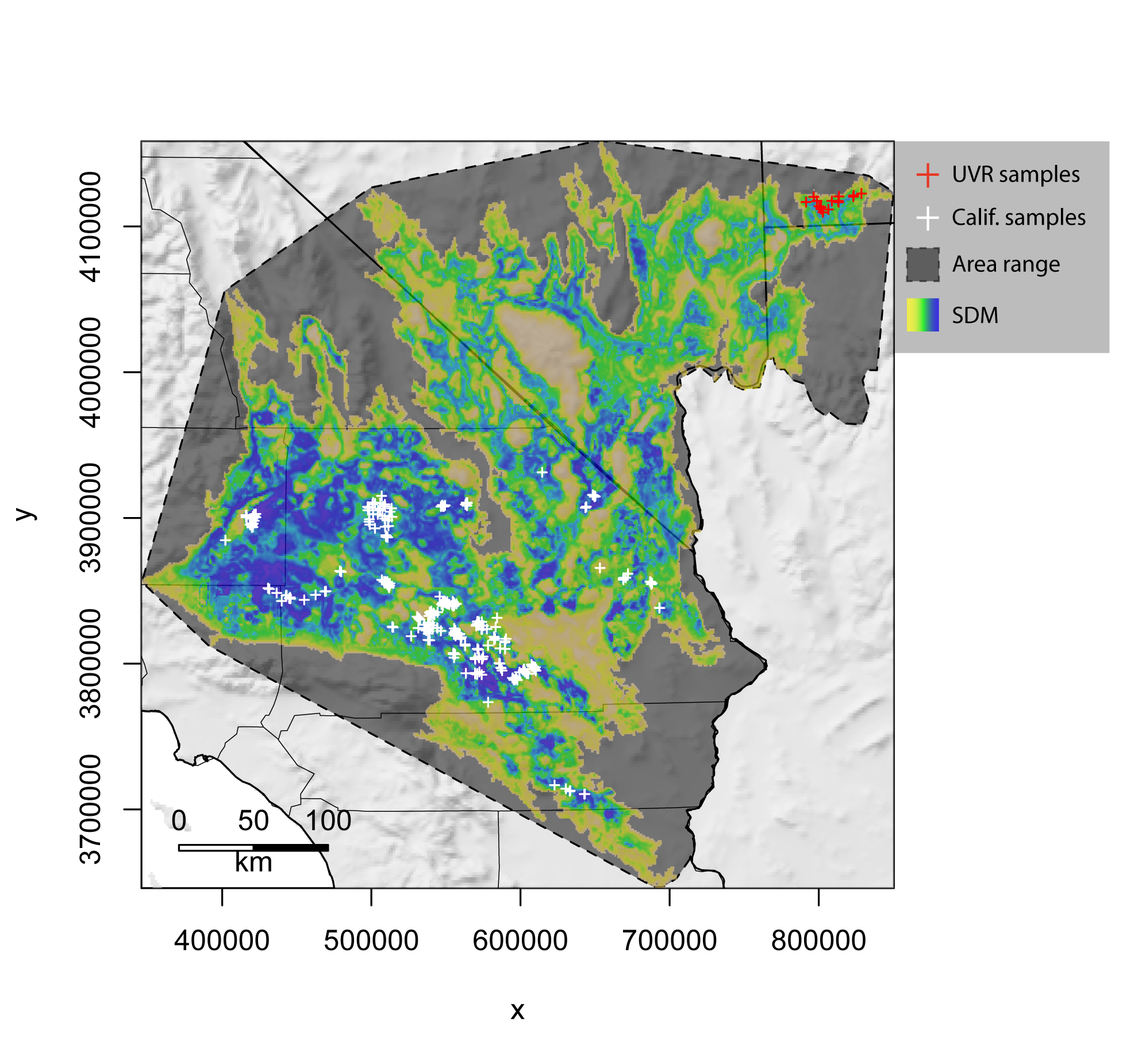
**
